# Patterns of chromosome 18 loss of heterozygosity in multifocal ileal neuroendocrine tumors

**DOI:** 10.1002/gcc.22850

**Published:** 2020-04-27

**Authors:** Zhouwei Zhang, Netta Mäkinen, Yosuke Kasai, Grace E. Kim, Begoña Diosdado, Eric Nakakura, Matthew Meyerson

**Affiliations:** ^1^ Department of Medical Oncology Dana‐Farber Cancer Institute Boston Massachusetts USA; ^2^ Cancer Program Broad Institute of Harvard and MIT Cambridge Massachusetts USA; ^3^ Department of Surgery University of California San Francisco San Francisco California USA; ^4^ Department of Pathology University of California San Francisco San Francisco California USA; ^5^ Division of Molecular Carcinogenesis Oncode Institute, The Netherlands Cancer Institute Amsterdam The Netherlands; ^6^ Departments of Genetics and Medicine Harvard Medical School Boston Massachusetts USA

**Keywords:** chromosome 18, copy number variation, high‐throughput sequencing, ileal neuroendocrine tumor, loss of heterozygosity

## Abstract

Ileal neuroendocrine tumors (NETs) represent the most common neoplasm of the small intestine. Although up to 50% of patients with ileal NETs are diagnosed with multifocal disease, the mechanisms by which multifocal ileal NETs arise are not yet understood. In this study, we analyzed genome‐wide sequencing data to examine patterns of copy number variation in 40 synchronous primary ileal NETs derived from three patients. Chromosome (chr) 18 loss of heterozygosity (LOH) was the most frequent copy number alteration identified; however, not all primary tumors from the same patient had evidence of this LOH. Our data revealed three distinct patterns of chr18 allelic loss, indicating that primary tumors from the same patient can present different LOH patterns including retention of either parental allele. In conclusion, our results are consistent with the model that multifocal ileal NETs originate independently. In addition, they suggest that there is no specific germline allele on chr18 that is the target of somatic LOH.

## 1 INTRODUCTION

Small intestinal neuroendocrine tumors (SI‐NETs) represent the most common neoplasm of the small bowel, accounting for 40% of all small intestinal malignancies.^1^ The estimated annual age‐adjusted incidence is 1.05/100 000 persons in the United States and has increased approximately 4‐fold since 1973.^2^, ^3^ SI‐NETs are generally slow‐growing, well‐differentiated tumors that are mostly found in the terminal ileum (ileal NETs), but show often high metastatic potential. About 50% of the patients have distant metastases at diagnosis, and the 5‐year survival rate for those patients is around 50%.^3^, ^4^, ^5^ For patients with metastatic disease, effective treatment modalities are limited.^4^

Previous high‐throughput sequencing studies have revealed that ileal NETs have a low somatic mutation rate.^6^, ^7^ To date, the most frequent genomic event identified in these tumors is loss of heterozygosity (LOH) at chromosome (chr) 18, occurring in approximately 60% of cases, and the only recurrent mutations identified to date are loss‐of‐function mutations in cyclin‐dependent kinase inhibitor 1B in 8% to 10% of tumors.^6^, ^7^, ^8^ Indeed, in a recent whole genome sequencing study of 2520 metastatic cancer/normal pairs, SI‐NETs were found to be the one tumor type in which a candidate driver gene was rarely found; 18 of 37 SI‐NETs had no identified candidate driver compared to 16 of 2483 other cancers.^9^

The majority of previous studies have focused on single primary tumors from each patient. However, up to 50% of patients with ileal NETs are diagnosed with multiple synchronous primary tumors.^10^, ^11^, ^12^ The molecular genetic mechanisms by which these multifocal lesions develop remain mostly unknown. One hypothesis is that the multifocal tumors may arise as a result of an unrecognized germline mutation.^13^ By analyzing LOH patterns at six candidate genetic loci using PCR‐based method, Katona et al. have suggested multifocal ileal NETs may originate independently.^14^ We report here the first high‐throughput sequencing‐based copy number profiling of multifocal primary ileal NETs. We not only confirm that chr18 LOH is the most common somatic copy number change in multifocal ileal NETs, but also reveal distinct patterns of chr18 allelic loss in individual tumors from the same patient. Our results corroborate the hypothesis that multiple primary ileal NETs may develop independently and provide novel insights into the molecular mechanism of chr18 LOH.

## 2 MATERIALS AND METHODS

### 2.1 Patients and tissue samples

**FIGURE 1:**
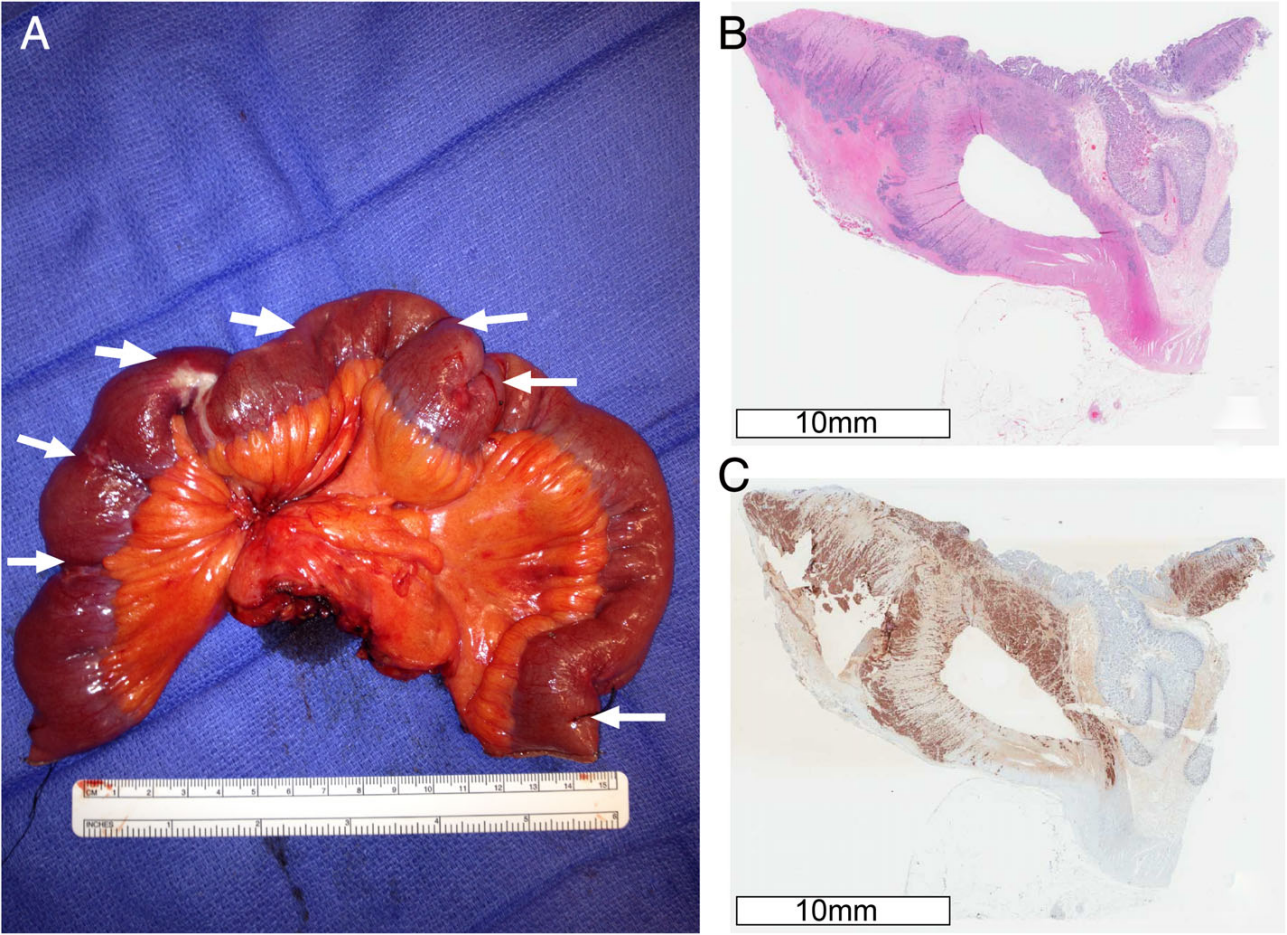
Representative images of multifocal ileal NETs from patient 1. A, Resected segment of ileum, showing multifocal NETs indicated by white arrows. B, Hematoxylin and eosin, and C, Chromogranin A staining of representative primary tumor tissue to confirm its neuroendocrine origin

Our sample set consisted of 40 de‐identified synchronous primary tumors and matched adjacent normal ileal mucosa specimens from three ileal NET patients (Figure 1; Table S1). A piece of each tissue sample was obtained, freshly frozen in liquid nitrogen, and stored at ‑80°C. All tumor specimens were stained with hematoxylin and eosin (H&E) and the presence and density of tumor cells were verified before sequencing. Each patient provided informed consent in accordance with the protocols approved by the Institutional Review Boards of the Dana‐Farber Cancer Institute and the University of California San Francisco.

### 2.2 Whole‐exome and linked‐read whole‐genome sequencing

Genomic DNA (gDNA) was extracted from fresh frozen tumor and adjacent normal ileum samples. Tissue specimens from patient 1 were sequenced using the 10× Genomics linked‐read WGS approach (10× Genomics, Pleasanton, California), as detailed before.^15^ Briefly, gDNA libraries were sequenced on an Illumina HiSeq platform to generate 151‐bp paired‐end reads with a mean depth of 60× coverage for tumor specimens, and 30× coverage for the tumor adjacent normal tissue. Whole‐exome sequencing (WES) was performed for patients 2 and 3, as previously described.^16^ In brief, exonic sequences were enriched using the Agilent V2 capture probe set (Agilent, Santa Clara, California) and sequenced by 76‐bp paired‐end reads using the Illumina Genome Analyzer IIx system (Illumina, San Diego, California) with a mean coverage of 80× for each base. Sequencing reads were aligned to the human reference genome GRCh37 (hg19).

### 2.3 Copy number variation and chr18 LOH mapping

For patient 1, somatic copy number changes and LOH in tumor samples were detected using a modified TITAN pipeline (https://github.com/gavinha/TitanCNA_10X_snakemake),^15^ and for patients 2 and 3, using CapSeg, allelic CapSeg, and ABSOLUTE algorithms in the Clonal Evolution Exome Suite.^17^, ^18^

Detailed LOH mapping was performed for tumors showing chr18 LOH by completing the following steps: (a) identifying germline heterozygous SNPs in the normal samples using the following filters: read depth >10 and variant allele frequency between 0.4 and 0.6^19^; (b) retrieving the allelic depth of the SNPs in the corresponding tumor samples if the total read depth of a given SNP is >10 in the tumor samples; (c) applying a binomial test to the read counts of the reference and alternative alleles of each SNP with the null hypothesis of 0.5, meaning that both alleles are expected to occur in half of the reads; (d) preserving SNPs with FDR‐adjusted *P* < 0.1 as LOH‐informative SNPs; (e) retaining tumor samples that have at least 1000 LOH‐informative SNPs (WGS, patient 1) or at least 100 LOH‐informative SNPs (WES, patient 2 and 3) for further analysis; (f) assigning the retained allele of each LOH‐informative SNP by comparing read counts of the reference and alternative alleles (Table S2).

## 3 RESULTS

### 3.1 Chr18 LOH is common in multifocal ileal NETs

Copy number analysis identified hemizygous loss of chr18 (log2 [copy number/2] < ‑0.1) to be the most common genomic alteration in multifocal ileal NETs (Figure S1), consistent with previous reports.^6^, ^8^ The frequency of chr18 LOH was 55% (6 of 11 primary tumors) in tumors from patient 1, 39% (7 of 18 primary tumors) in tumors from patient 2, and 27% (3 of 11 primary tumors) in tumors from patient 3. Other chromosomal copy number changes included gains of chromosomes 4, 5, 7, 14, and 20; none of these changes were present in tumors from more than one patient.

### 3.2 Multifocal ileal NETs show different patterns of allelic loss at chr18

**FIGURE 2:**
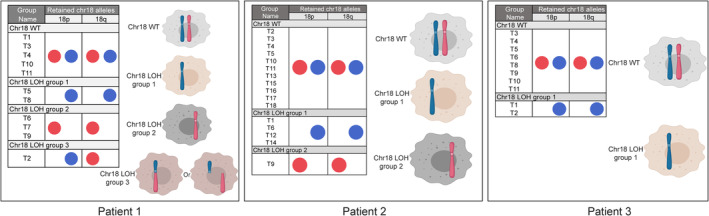
Schematic representation of chr18 LOH patterns occurring in multifocal tumors from three ileal NET patients. Red and blue dots in figure inset represent parental chr18 alleles

By comparing retained SNP alleles between tumor samples with chr18 LOH, we observed three different chr18 LOH patterns in multifocal ileal NETs. Individual tumors with chr18 allelic loss from the same patient can lose either (a) the same parental allele, (b) different parental alleles with consistent allelic loss across chromosomal arms, or (c) different parental alleles in the short (p) and long (q) arms of chr18 (Figure 2). Copy number analysis of patient 1 identified six primary ileal NETs displaying chr18 LOH. Based on 359 common LOH‐informative SNPs, two of the tumors (T5 and T8) had lost the same parental allele, while three of the tumors (T6, T7, and T9) had lost the other parental allele (Figure 2; Tables S3 and S4). Both groups had consistent allelic loss patterns across both chromosomal arms. Interestingly, one tumor (T2) from patient 1 had lost different parental alleles in the p and q arms of chr18. In other words, the lost allele of T2 in 18p was the same as that of T5 and T8, while the allele of T2 lost in 18q was the same as that of T6, T7, and T9 (Figure 2; Tables S3 and S4). For patient 2, five out of seven tumors with chr18 LOH were included in LOH mapping. By comparing retained alleles of 116 common LOH‐informative SNPs, four of the five tumors had lost the same parental allele of the whole chr18 (T1, T6, T12 and T14), while one tumor (T9) had lost the other parental allele of the whole chr18 (Figure 2; Tables S3 and S5). Lastly, two out of three primary tumors displaying chr18 LOH from patient 3 (T1 and T2) were analyzed and found to have lost the same parental allele (Figure 2; Tables S3 and S6).

## 4 DISCUSSION

Ileal NETs represent the most common neoplasm of the small intestine with a high incidence of multiple synchronous primary tumors at diagnosis. Our understanding of the tumor multifocality, however, remains limited with respect to the molecular mechanisms underlying their tumorigenesis and effects on clinical management.^11^, ^12^, ^20^ Here, we utilized high‐throughput sequencing to characterize copy number alterations present in multifocal ileal NETs to better understand the molecular genetic background of these lesions.

Most large‐scale sequencing studies to date have concentrated on sequencing single primary tumors from ileal NET patients.^6^, ^7^ This is, to our knowledge, the first comprehensive copy number profiling study of multifocal primary ileal NETs. The observed copy number changes included loss of chr18 and gains of chr4, chr5, chr7, chr14, and chr20. These findings are in agreement with what others and we have previously reported when studying single primary ileal NETs from different individuals.^6^, ^7^, ^21^ More importantly, our data corroborate that chr18 loss is the most prominent copy number alteration in ileal NETs, regardless of whether tumors are unifocal or multifocal. Of note, not all primary tumors from the same patient displayed chr18 loss.

The origin of multifocal ileal NETs is not well understood. Previous studies have proposed that multifocal ileal NETs may arise due to germline predisposition or as independent clones.^13^, ^14^ Sei et al. used WES to screen for single nucleotide germline variants in 33 SI‐NET families with multiple synchronous tumors but was unable to identify a common germline mutation in these families.^13^ By analyzing LOH patterns at six polymorphic microsatellite loci on four different chromosomes in multifocal SI‐NETs, Katona et al. concluded that most synchronous tumors are likely to arise independently.^14^ In our study, we analyzed high‐throughput sequencing data to determine the patterns of chr18 allelic loss in multifocal ileal NETs, which can be used to infer the clonal relationship between individual tumors. Intriguingly, different LOH patterns were observed. Tumors with chr18 LOH from patients 1 and 2 displayed mixed LOH patterns, losing either one or the other parental allele, while two primary tumors showing chr18 LOH from the patient 3 lost the same chr18 parental allele. The LOH patterns for all patients were consistent throughout both arms of chr18 with one exception: one tumor from patient 1 was observed to lose different parental alleles at 18p and 18q.

The significance of our study is three‐fold. First, consistent with previous results, we show that chr18 loss occurs in many, but not all, primary ileal NETs.^6^, ^8^ Second, we demonstrate that primary ileal NETs display mixed allelic loss patterns in chr18 among individual tumors from the same patient. These results suggest that multifocal ileal NETs are likely to be polyclonal, which is largely in line with the findings of Katona et al.^14^ Meanwhile, our analysis addresses two limitations from the earlier study,^14^ which we were able to overcome thanks to advances in genome analysis technology during the intervening years. Katona et al. only assessed LOH patterns for two microsatellite loci on the 18q arm, which might not reflect LOH patterns across the entire chr18, while we mapped LOH patterns across the whole chromosome. Furthermore, Katona et al. used a less sensitive PCR‐based genotyping method, whereas we refined the detection by conducting high‐throughput sequencing. Third, given the frequent chr18 LOH in ileal NETs, it would be reasonable to infer that the reason for chr18 LOH is to delete a wild type copy of a tumor suppressor gene in the setting of a germline loss‐of‐function mutation. However, the loss of either parental allele, in tumors from the same patient, rules out this possibility. Thus, our data suggest that it is improbable that there exists a germline loss‐of‐function mutation in a tumor suppressor gene on chr18. Rather, we conclude that it is more likely that the oncogenic effect of chr18 allelic loss may be due to a dosage impact on the expression of one, several or many genes within the chromosome.

There are some limitations in our current study. First, our interpretation of LOH pattern assumed that the hemizygous loss of chr18 in ileal NET is more likely to occur in a single parental allele as previously reported.^6^, ^7^, ^22^, ^23^ Tissue karyotyping and fluorescence in situ hybridization analysis may be required for further confirmation. Second, we could not map the LOH pattern for all tumors displaying chr18 LOH because of a small number of analyzable LOH‐informative SNPs found in some tumors. This may be explained by a lower amount of tumor cells in those tissue samples. Last, our current data were not able to pinpoint the exact mechanism of how a tumor (T2 from patient 1) lost different parental alleles in p and q arms of chr18. One possibility is that homologous recombination occurs at chr18, and one of the recombined copies is lost during tumorigenesis. Alternatively, this tumor might undergo loss of the two arms of chr18 separately, indicating that the chr18 LOH of ileal NETs may represent two independent genomic events (Figure 2). Harnessing long‐read sequencing technologies to generate ultra‐long reads that cover both chromosomal arms may be required to reveal the exact mechanism.^24^, ^25^

In conclusion, our data demonstrate that chr18 LOH is commonly found in multifocal ileal NETs. The distinct LOH patterns in individual tumors from the same patients suggest that there is not a particular germline allele on chr18 that predisposes somatic LOH.

## CONFLICT OF INTEREST

The authors declare that they have no conflicts of interest with the contents of this article. However, Dr Meyerson declares the following general conflicts of interest: research support from Bayer, Ono, Novo, and Janssen; patent licensing royalties from LabCorp; and serving as scientific advisory board chair and consultant for OrigiMed.

## Supporting information


**Figure S1** Copy number profiles of all primary synchronous ileal NETs

**Table S1** Patient information

**Table S2** Summary of SNP filtering for LOH mapping

**Table S3** Summary table of chr18 LOH patterns for three patients

**Table S4** Retained alleles of annotated SNPs of tumors displaying chr18 LOH in patient 1

**Table S5** Retained alleles of annotated SNPs of tumors displaying chr18 LOH in patient 2

**Table S6** Retained alleles of annotated SNPs of tumors displaying chr18 LOH in patient 3

## Data Availability

The data that support the findings of this study are currently being submitted to the European Genome‐phenome Archive.
