# Supplementary figures and images for: Patterns of chromosome 18 loss of heterozygosity in multifocal ileal neuroendocrine tumors
Source: Genes Chromosomes Cancer. 2020 Apr 27;59(9):535–9. doi: 10.1002/gcc.22850 (PMC7384092; doi:10.1002/gcc.22850)

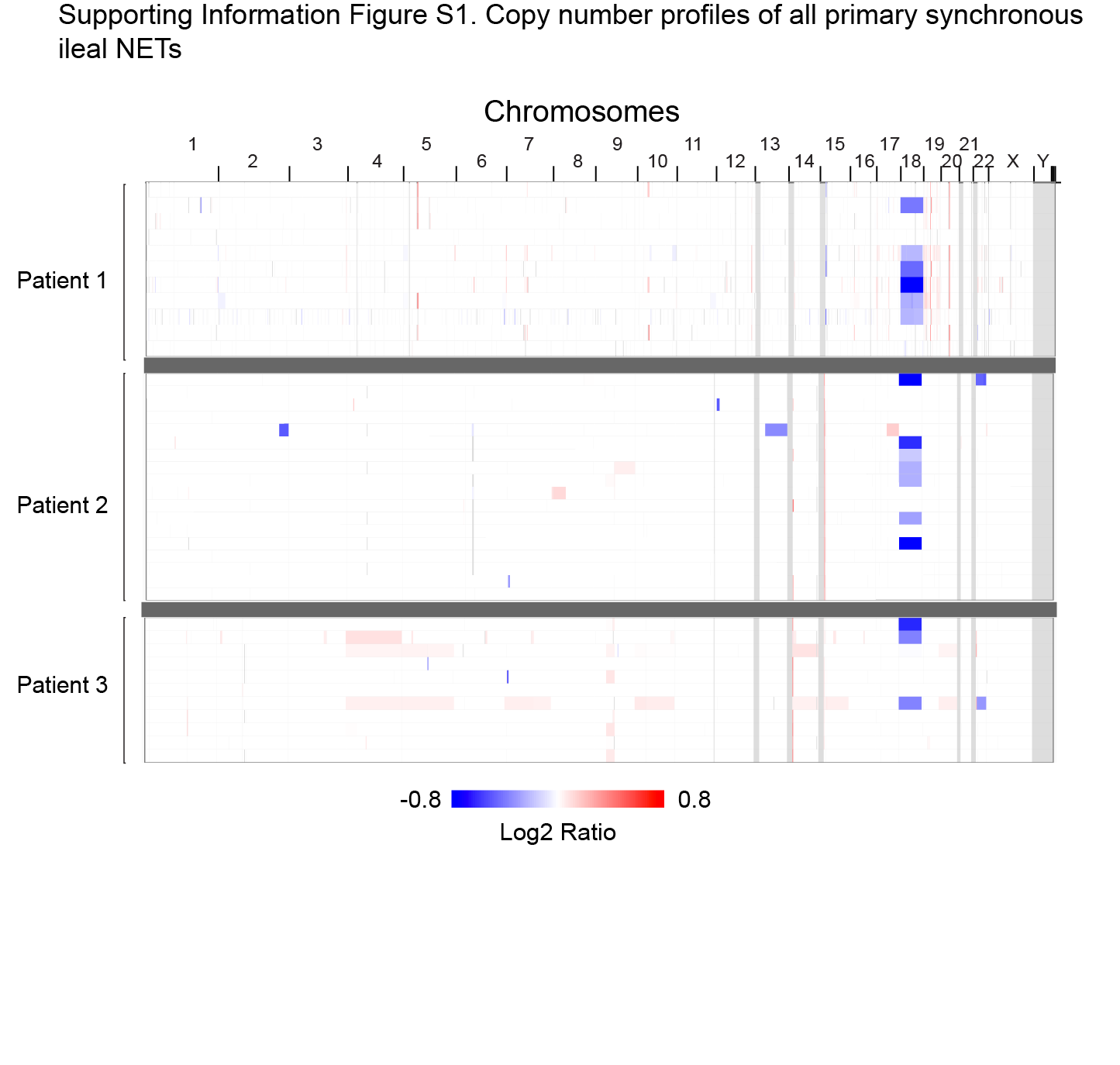

Supplement: Supplementary file 1 — Figure S1 Copy number profiles of all primary synchronous ileal NETs [file GCC-59-535-s001.tif]
