# Supplementary material for: Patterns of chromosome 18 loss of heterozygosity in multifocal ileal neuroendocrine tumors
Source: Genes Chromosomes Cancer. 2020 Apr 27;59(9):535–9. doi: 10.1002/gcc.22850 (PMC7384092; doi:10.1002/gcc.22850)
Supplement: Supplementary file 2 — Table S1 Patient information [file GCC-59-535-s002.docx]

Supporting Information Table S1. Patient information

| Patient | Age | Gender | # Primary tumors | Max size | T* | N* | M* | Stage* | Grade† |
| --- | --- | --- | --- | --- | --- | --- | --- | --- | --- |
| 1 | 63 | F | 11 | 2.9 cm | T3 | N1 | M1a | IV | G1 |
| 2 | 63 | M | 18 | 2.0 cm | T3 | N2 | M1c | IV | G1 |
| 3 | 56 | M | 11 | 1.1 cm | T3 | N2 | M0 | III | G2 |

* TNM staging was based on American Joint Committee on Cancer 8th edition.

† Grade was based on WHO Classification 5th edition.
