# Supplementary material for: Patterns of chromosome 18 loss of heterozygosity in multifocal ileal neuroendocrine tumors
Source: Genes Chromosomes Cancer. 2020 Apr 27;59(9):535–9. doi: 10.1002/gcc.22850 (PMC7384092; doi:10.1002/gcc.22850)
Supplement: Supplementary file 3 — Table S2 Summary of SNP filtering for LOH mapping [file GCC-59-535-s003.docx]

Supporting Information Table S2. Summary of SNP filtering for LOH mapping

|  | Patient 1 | Patient 2 | Patient 3 |
| --- | --- | --- | --- |
| Sequencing method | WGS | WES | WES |
| # Chr18 LOH tumors | 6 | 7 | 3 |
| # Total SNPs (rs coded) | 50798 | 633 | 647 |
| # Common SNPs after depth and | 21462 | 308 | 372 |
| normal heterozygous sites filters* |  |  |  |
| # Chr18 LOH tumors after filtering for | 6 | 5 | 2 |
| LOH-informative SNPs† |  |  |  |
| # Common LOH-informative SNPs | 359 | 116 | 119 |
| (FDR < 0.1) |  |  |  |

* SNP sites were filtered by read depth >10 and variant allele frequency between 0.4 and

0.6 in germline sample.

† Tumor sample was included if it had at least 1000 (WGS, patient 1) or 100 (WES, patient 2

and 3) LOH-informative SNPs after binomial test and FDR correction.
