# Supplementary material for: Patterns of chromosome 18 loss of heterozygosity in multifocal ileal neuroendocrine tumors
Source: Genes Chromosomes Cancer. 2020 Apr 27;59(9):535–9. doi: 10.1002/gcc.22850 (PMC7384092; doi:10.1002/gcc.22850)
Supplement: Supplementary file 4 — Table S3 Summary table of chr18 LOH patterns for three patients [file GCC-59-535-s004.docx]

Supplementary Information Table S3. Summary table of chr18 LOH patterns for three patients

|  | **p arm** | | **q arm** | |
| --- | --- | --- | --- | --- |
| Chr18 LOH | Allele 1 | Allele 2 | Allele 1 | Allele 2 |
| **Patient 1** |  |  |  |  |
| Group1 | 95/97 (98%) |  | 248/262 (95%) |  |
| Group2 |  | 91/97 (94%) |  | 251/262 (96%) |
| Group3 | 90/97 (93%) |  |  | 245/262 (94%) |
| **Patient 2** |  |  |  |  |
| Group 1 | 19/19 (100%) |  | 97/97 (100%) |  |
| Group 2 |  | 19/19 (100%) |  | 97/97 (100%) |
| **Patient 3** |  |  |  |  |
| Group 1 | 34/34 (100%) |  | 85/85 (100%) |  |
